# STAT3 Inhibition to Treat Ulcerative Colitis-Associated Colorectal Cancer

**DOI:** 10.3390/ijms262110808

**Published:** 2025-11-06

**Authors:** Prema Robinson, Zal Italia, Zara Italia, Tan Hoang, Emma Rodriguez, T. Kris Eckols, Moses Kasembeli, Leticia Hamana Zorrilla, Luisa Maren Solis Soto, Rajasekaran Mahalingam, David J. Tweardy

**Affiliations:** 1 Departments of Infectious Diseases, Infection Control & Employee Health, Division of Internal Medicine, The University of Texas MD Anderson Cancer Center, Houston, TX 77030-4009, USA; zsitalia1@mdanderson.org (Z.I.); italia.zara@yahoo.com (Z.I.); tmhoang@mdanderson.org (T.H.); eerodriguez1@mdanderson.org (E.R.); kriseckols@gmail.com (T.K.E.); mmkasembeli@mdanderson.org (M.K.); 2 Department of Translational Molecular Pathology, The University of Texas MD Anderson Cancer Center, Houston, TX 77030-4009, USA; lghamana@mdanderson.org (L.H.Z.); lmsolis@mdanderson.org (L.M.S.S.); 3 Department of Symptom Research Biology, The University of Texas MD Anderson Cancer Center, Houston, TX 77030-4009, USA; rmahalingam@mdanderson.org; 4 Department of Molecular & Cellular Oncology, The University of Texas MD Anderson Cancer Center, Houston, TX 77030-4009, USA

**Keywords:** colorectal cancer, cancer treatment, STAT3, inflammatory bowel disease

## Abstract

In patients with inflammatory bowel disease (IBD), colorectal cancer (CRC) occurs with 20-to-30-fold higher frequency, is more advanced at diagnosis, and has a worse prognosis than in the general population. To improve their treatment options, we determined if targeting STAT3 with TTI-101, a small-molecule STAT3 inhibitor, was beneficial in the azoxymethane (AOM)-disodium sulfate (DSS) mouse model of colitis-associated CRC. C57BL/6 mice received a single intraperitoneal injection of AOM followed by three cycles of 5% DSS in drinking water before receiving TTI-101 (50 mg/kg by oral gavage, OG, and daily) or vehicle for 28 days. TTI-101 treatment reduced adenoma numbers by 89% from 1.14 ± 1.07 in vehicle-treated mice to 0.13 ± 0.35 in TTI-101-treated mice (*p* ≤ 0.05, Kruskal–Wallis test). Levels of activated STAT3 (pY-STAT3) were increased 3.3-fold in the epithelium and stroma of dysplastic mucosa (147 ± 46; mean ± SD; and n = 4) vs. normal mucosa (45 ± 26; n = 7; and *p* ≤ 0.05, Kruskal–Wallis test) and were correlated with the adenoma number. TTI-101 was detected at pharmacologically relevant levels in the plasma and colons of TTI-101-treated AOM-DSS mice and was concentrated within colon tissue; plasma TTI-101 levels inversely correlated to pY-STAT3 levels. Importantly, TTI-101 normalized the colon transcriptome of AOM-DSS mice and reduced the expression of STAT3- and STAT1-upregulated genes associated with CRC oncogenesis. Thus, TTI-101 treatment may benefit IBD patients with CRC.

## 1. Introduction

Colorectal cancer is the second leading cause of cancer-related death in the United States, resulting in over 50,000 deaths in 2024 [1]. The lifetime risk of developing CRC in the general population is ~5%. However, in certain individuals, including patients with inflammatory bowel disease (IBD), especially ulcerative colitis (UC), the risk of CRC is 20-to 30-fold higher than in the general population [2]. CRC in IBD patients is also more advanced at diagnosis and is accompanied by greater mortality [3].

CRC arises in IBD with a background of chronic inflammation within the intestinal stromal cell compartment [4] mediated, in part, by group 3 innate lymphoid cells (ILC3) that produce interleukin (IL)-22 and IL-17, leading to mucosal inflammation, epithelial barrier disruption, and the recruitment of inflammatory myeloid cells [5,6]. These cytokines, along with IL-6, the pro-inflammatory cytokine most often invoked in the pathogenesis of IBD and IBD-associated CRC, signal through the signal transducer and activator of transcription (STAT) 3. Evidence has accumulated that STAT3 contributes to sporadic colorectal cancer [7,8], as well as to CRC in IBD patients, who are already at an increased risk for CRCSTAT3 protein levels are increased in 52–73% of human CRC tumors and levels of STAT3 correlated with the depth of tumor invasion, venous invasion, lymph node metastasis, and CRC-specific mortality [7,8]. Mice expressing only the pro-inflammatory STAT3α isoform developed colon tumors earlier in the azomethane (AOM) + dextran sodium sulfate (DSS) model of colitis-associated CRC than wild type mice [9]. In other studies using the AOM-DSS model, mice deficient in STAT3 in their intestinal epithelial cells demonstrated reduced tumor size and reduced tumor incidence [10].

Our group developed TTI-101, a small-molecule STAT3 inhibitor, that targets the phosphotyrosyl (pY) peptide-binding pocket within the STAT3 SH2 domain and blocks binding to its pY-peptide ligand within cytokine-activated receptors [11], thereby inhibiting cytokine-mediated STAT3 activation [i.e., reducing the phosphorylation of tyrosine (Y) 705, pY-STAT3]. Here, we examined the ability of TTI-101 to treat CRC in the AOM-DSS mouse model of colitis-induced CRC and demonstrated that TTI-101 markedly reduced colon tumors in this model, at least in part, by normalizing the STAT3-driven pro-oncogenic transcriptome in colonic mucosa.

## 2. Results

### 2.1. TTI-101 Reduced Adenoma Numbers in the AOM-DSS Mouse Model of Colitis-Induced CRC and Was Well Tolerated

**Figure 1:**
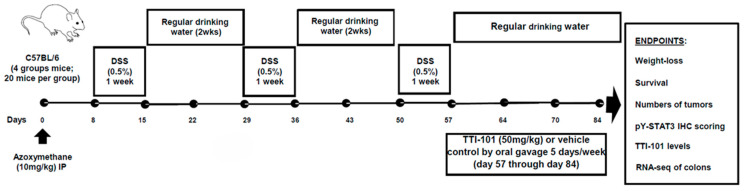
Schema of AOM-DSS mouse model of colitis-induced CRC. C57BL/6 were entered into the AOM-DSS model of colitis-induced CRC and randomized into four groups (20 mice per group) to receive AOM, DSS, TTI-101, or vehicle control, as indicated. One group of mice was given AOM alone and treated with vehicle control; one group of mice was given AOM alone and treated with TTI-101 (50 mg/mL); one group of mice was given AOM plus DSS and treated with vehicle control; and one group mice was given AOM plus DSS and treated with TTI-101. Mice were evaluated for weight loss and survival from day 1 to day 84 and euthanized on day 84. Their colons were examined grossly for numbers of polyps and microscopically for adenomas and/or adenocarcinomas; colons and plasma were examined further, as indicated.

**Figure 2:**
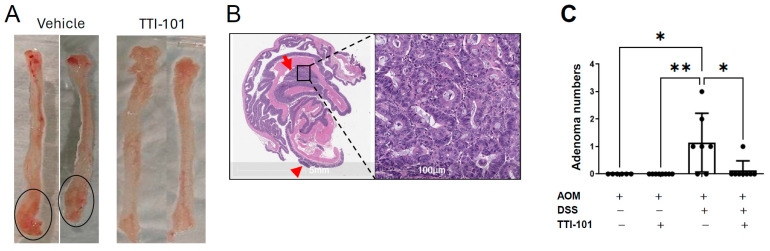
Colon adenoma numbers in the AOM-DSS mouse model of colitis-induced CRC and the effect of TTI-101 treatment. Photographs of representative colons (**A**) from two vehicle-treated AOM-DSS mice (left panel) and from two TTI-101-treated AOM-DSS mice (right panel; polyps circled in black). Photomicrograph (**B**) of a representative H&E section of a Swiss-rolled colon (5×; left panel) from a vehicle-treated AOM-DSS mouse showing two adenomas (red arrows); a magnified image of one of the adenomas is shown (200×; right panel). Adenoma numbers (**C**) in colons of mice treated as indicated (data shown are mean ± SD; * *p* ≤ 0.05; ** *p* ≤ 0.01; and Kruskal–Wallis test).

We counted numbers of colon polyps, measured body weights, and assessed survival in four groups of C57BL/6 mice subjected to the AOM-DSS model of colitis-induced CRC (Figure 1). Groups 1 and 2 received AOM alone and were treated with either vehicle control or TTI-101 (50 mg/mL); Groups 3 and 4 received AOM plus DSS and were treated with either vehicle control or TTI-101. There were no polyps in either of the AOM alone groups. However, five of seven vehicle-treated AOM-DSS mice had polyps (1.14 ± 1.07, mean ± SD; Figure 2A,C) that proved to be adenomas on histopathological examination (Figure 2B). In contrast, only one of eight TTI-101-treated AOM-DSS mice had a polyp/adenoma (0.13 ± 0.35; *p* ≤ 0.05, Kruskal–Wallis test). We did not observe any toxicity or differences in body weights and survival between the TTI-101-treated and vehicle-treated mice; thus, TTI-101 was well tolerated. TTI-101 treatment markedly decreased adenoma numbers in the AOM-DSS model of UC-induced CRC.

### 2.2. Levels of pY-STAT3 Were Increased in Dysplastic vs. Normal Colon Mucosa of AOM-DSS Mice and Correlated with Adenoma Number

**Figure 3:**
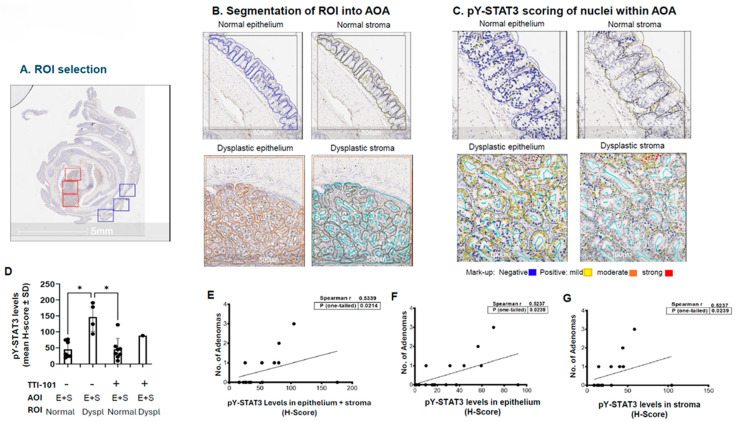
Location and quantification of pY-STAT3 in colons of AOM-DSS mice and their relationship to adenoma numbers. Regions of interest (ROI) within Swiss-rolled colons (**A**) were selected within areas of normal colonic mucosa (blue) and dysplastic colonic mucosa (red; 3–5 ROI each). Normal and dysplasia ROI were segmented into two areas of analysis (AOA)—epithelium and stroma (**B**)—by outlining them manually or using annotation tools within the imaging software. Normal epithelium is outlined in blue; normal stroma is outlined in yellow; dysplastic epithelium is outlined in orange; and dysplastic stroma is outlined in cyan. Nuclei (~100) within each AOA were scored for level of pY-STAT3 and color coded (**C**)—negative staining (blue; score = 0) and positive staining with three intensities—mild (yellow; score = 1), moderate (orange; score = 2), and strong (red; score = 3). The pY-STAT3 H-scores were tallied for nuclei within epithelium plus stroma in normal and dysplastic mucosa of AOM-DSS vehicle- and TTI-101-treated mice and are shown ((**D**); mean ± SEM; * *p* < 0.05; and Student’s *t*-test). Adenoma numbers in the combined vehicle- and TTI-101-treated AOM-DSS mice correlated with pY-STAT3 H scores within epithelium plus stroma (**E**), epithelium alone (**F**), and stroma alone ((**G**); all, *p* ≤ 0.05, and Spearman).

The results above suggested that levels of activated (pY)-STAT3 were increased in dysplasia vs. normal colonic mucosa and that TTI-101 decreased adenomas by reducing the levels of pY-STAT3 in dysplastic mucosa. To examine these hypotheses, we performed pY-STAT3 IHC staining of sections of Swiss-rolled colons from vehicle- and TTI-101-treated AOM-DSS mice; digital image analysis was used to assess levels of nuclear pY-STAT3 within the epithelial and stromal cells of normal vs. dysplastic colonic mucosa (Figure 3). Levels of nuclear pY-STAT3 were increased 3.3-fold in the epithelium and stroma (E+S) of dysplastic mucosa (147 ± 46; mean ± SD) vs. normal mucosa (45 ± 26; *p* ≤ 0.05, Kruskal–Wallis test; Figure 3D); this increase also occurred in stromal cells alone (65 ± 14, dysplastic mucosa vs. 22 ± 11, normal mucosa; *p* ≤ 0.05, Kruskal–Wallis test), in epithelial cells alone (81 ± 32, dysplastic mucosa vs. 24 ± 17, normal mucosa; however, the increase did not achieve significance; and *p* = 0.0596, Kruskal–Wallis test); and in mucosa alone. Importantly, the levels of nuclear pY-STAT3 were lower in epithelial and stromal cells combined, epithelial cells alone, and stromal cells alone within the normal mucosa of TTI-101-treated mice vs. dysplastic mucosa of vehicle-treated mice (all; *p* ≤ 0.05, Kruskal–Wallis test; Figure 3D. Furthermore, the number of colon adenomas correlated with pY-STAT3 levels in the epithelium plus stroma, as well as with pY-STAT3 levels in the epithelium alone and in the stroma alone (Figure 3E–G).

### 2.3. TTI-101 Was Detected at Pharmacologically Relevant Levels in Plasma and Colons of TTI-101-Treated AOM-DSS Mice; TTI-101 Levels Were Inversely Correlated to pY-STAT3 Levels

**Figure 4:**
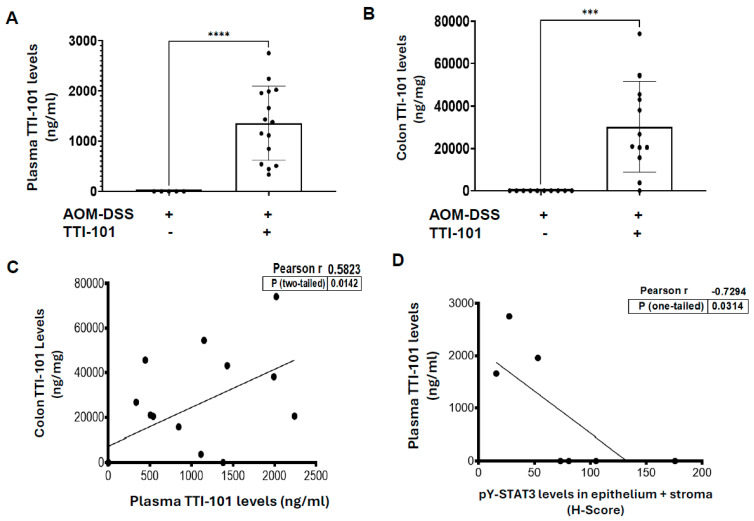
TTI-101 was detected at pharmacologically relevant levels in plasma and colons of TTI-101-treated AOM-DSS mice. TTI-101 levels in the plasma (**A**) and colon (**B**) from vehicle-treated and TTI-101-treated AOM-DSS mice are shown (mean ± SEM; ****, *** *p* < 0.001; and Student’s *t* test). TTI-101 levels in the colon correlated with TTI-101 levels in the plasma ((**C**); *p* ≤ 0.05, Pearson); plasma TTI-101 levels were inversely correlated with pY-STAT3 H scores in epithelium plus stroma ((**D**); *p* ≤ 0.05, Pearson).

TTI-101 was detected in the plasma of all TTI-101-treated mice and in the colons of all but one TTI-101-treated mouse (Figure 4A,B); TTI-101 was not detected in the plasma or colons of TTI-101-untreated mice. Colon levels of TTI-101 correlated positively with plasma levels of TTI-101 (Figure 4C; *p* ≤ 0.05, Pearson’s). In addition, the mean colon TTI-101 level was 14-fold greater than the IC_50_ for the inhibition of STAT3-driven proliferation [12]. Also of note, the TTI-101 levels in plasma inversely correlated with the levels of pY-STAT3 in the epithelium plus stroma (Figure 4D; *p* ≤ 0.05, Pearson’s). These results strongly suggest that TTI-101′s ability to reduce adenoma numbers in the colons of AOM-DSS mice is mediated pharmacologically through its ability to reduce pY-STAT3 levels in colonic mucosa.

### 2.4. Reduction in Adenomas by TTI-101 Is Accompanied by Normalization of the Colon Transcriptome of AOM-DSS Mice

**Figure 5:**
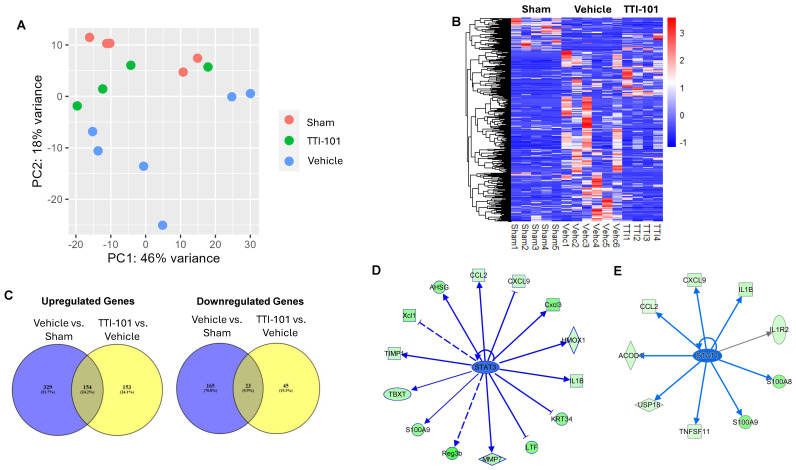
Effect on colon gene expression by adding DSS to AOM and by treating AOM-DSS mice with TTI-101. (**A**) Principal Component Analysis (PCA) mapping of aggregate gene expression profiles of individual mouse colons within the AOM alone group (Sham), the AOM-DSS vehicle-treated group (vehicle), and the AOM-DSS TTI-101-treated group (TTI-101). (**B**) Supervised hierarchical clustering and heat map showing relative mRNA expression levels in the mouse colons from the three groups. (**C**) Venn diagrams depicting the results of two comparisons of mRNA sets: vehicle-treated AOM-DSS (vehicle) vs. AOM alone mRNA sets (Sham; Comparison 1) and TTI-101-treated AOM-DSS (TTI-101) vs. AOM-DSS (vehicle) mRNA sets (Comparison 2); this depiction is focused on two groups of overlapping mRNAs within these two comparisons—those increased by DSS that were decreased by TTI-101 treatment (upregulated genes, 154 genes; (**C**), left panel) and those decreased by DSS that were increased by TTI-101 (downregulated genes, 23 genes (**C**); right panel). (**D**) Diagram of IPA of 19 STAT3-upregulated genes whose mRNA levels were increased by the addition of DSS to AOM and were also reduced by TTI-101 treatment. STAT3 in the center of diagram is shown in blue to indicate that it is increased by the addition of DSS and reduced by TTI-101 treatment. STAT3-upregulated gene targets that are decreased by TTI-101 are shown in green with their shapes corresponding to gene class as follows: square = cytokine, oval = transcriptional regulator, horizontal diagonal = peptidase, vertical diagonal = enzyme, and round = other. The blue line connectors between STAT3 and each of its targets (pointed or blunt arrowhead) indicate that the STAT3-upregulated gene target is decreased by TTI-101; solid lines indicate that the effect of STAT3 on the gene target results from a direct interaction, while a dashed line indicates that it results from an indirect interaction. (**E**) Diagram of IPA of 9 STAT1-upregulated genes whose mRNA levels were increased by the addition of DSS to AOM and reduced by TTI-101 treatment. The interpretation of the symbols, colors, and line formats are the same as in panel (**D**); the gray line indicates the effect of STAT1 on the gene target is undefined.

To examine the effect of TTI-101 on the colon transcriptome of AOM-DSS mice and to identify STAT3 gene targets that may mediate TTI-101′s ability to reduce adenomas, we performed transcriptome analyses on mouse colons from three groups—mice given AOM alone (Sham), vehicle-treated AOM-DSS mice (Vehicle), and TTI-101-treated AOM-DSS mice (TTI-101). A Principle Component Analysis (CPA) of the colon transcriptomes from mice in these three groups (Figure 5A) showed the following: (1) clustering of the Sham mouse colon transcriptomes along the PC2 axis; (2) separation of the vehicle mouse transcriptome cluster from the Sham mouse cluster along the PC2 axis; and (3) movement of the TTI-101 mouse cluster toward the Sham mouse cluster along the PC2 axis. These results indicate that there were substantial transcriptome changes induced by the addition of DSS to AOM and that these changes were in large measure reversed by TTI-101 treatment. These findings were reinforced by hierarchical clustering of the top 500 most variable DEGs across the three groups (Figure 5B).

To identify potential candidate mRNAs that contribute to adenoma formation in the AOM-DSS model and their reduction with TTI-101 treatment, we first performed two comparisons of mRNA sets: vehicle-treated AOM-DSS (vehicle) vs. AOM alone mRNA sets (Sham; Comparison 1) and TTI-101-treated AOM-DSS (TTI-101) vs. AOM-DSS (vehicle) mRNA sets (Comparison 2) and identified those genes that were differentially expressed (Figure 5C). Included in the comparisons were all coding and multiple complex genes that were present in all samples of both sets used in each comparison. The criteria for differential expressions were *p*-values ≤ 0.05 and a log 2-fold change of ≤−1 and ≥+1. We then focused on two groups of overlapping mRNAs within these two comparisons—those increased by the addition of DSS to AOM that were decreased by TTI-101 treatment (upregulated genes, 154 genes; Figure 5C, left panel) and those decreased by the addition of DSS to AOM that were increased by TTI-101 treatment (downregulated genes, 23 genes; Figure 5C, right panel); we performed ingenuity pathway analysis (IPA) on both groups of genes. Among the upregulated gene group of 154 genes, 19 genes were STAT3 gene targets (Table 1); the levels of expression of all 19 STAT3 upregulated genes were reduced by TTI-101 (Table 1 and Figure 5D). Notably, all 19 STAT3-upregulated genes have been associated with colorectal cancer, with 16 of these 19 genes shown to contribute to CRC progression and metastases, including CD244 [13], CXCL9 [14], LYZ2 [15], MMP7 [16], S100A9 [17], TIMP-1 [18], TNFS11 [19], XCL1 [20], AHSG [21], CCL17 [22], CCL2 [23], HMOX1 [24], IL1B [25], LEF1 [26], and USP18 [27]. The three remaining STAT3 upregulated genes that were decreased by TTI-101 have been shown to protect against CRC, including LTF [28], REGB [29], and SOX11 [30].

Interestingly, 9 of the 154 upregulated genes have been shown to be STAT1 gene targets; these include CCL2 [31], CXCL9 [32], IL1B [33], IL1R2 [34], S100A8 [35], TNFSF11 [36], USP18 [37], ACOD [38], and S100A9 [17]. Of these nine genes, six genes (CCL2, CXCL9, IL1B, TNFSF11, USP18, and S100A9) are also STAT3 gene targets, which leaves three genes as potentially regulated only by STAT1. The mRNA levels of these three upregulated genes were decreased in the colons of the TTI-101-treatment group (Figure 5E) and were shown by IPA to be associated with CRC progression, metastases, and poor prognosis.

Of the 23 downregulated genes (genes decreased by the addition of DSS to AOM that were increased by TTI-101 treatment), IPA revealed that these genes were not linked with CRC pathogenesis or needed hypermethylation to exert a role in CRC pathogenesis, including the top 10 genes that underwent the greatest change: LEP [39], RASGRF1 [40], Best4-ps [41], NSUN7 [42], OAZ3 [43], Maskbp3 [44], KCNS1 [45], LINGO1 [46], FLRT1 [47], and DNAH14 [48]. Taken together, the transcriptomic results indicate that there is a marked anti-oncogenic effect of TTI-101 on the CRC transcriptome mediated largely by its targeting of STAT3 and STAT1 in the colons of TTI-101-treated AOM-DSS mice, which helps to explain its ability to reduce colon adenomas (Figure 2).

## 3. Discussion

STAT3 has been implicated in IBD-associated CRC in patients; genetically engineered mouse models (GEMM) have also provided a compelling proof of concept for its contribution to CRC. However, the effect of targeting STAT3 on IBD-associated CRC has not been reported. In this paper, we demonstrated that TTI-101, a small-molecule STAT3 inhibitor currently under investigation in a three-arm, Phase II trial for treatment of hepatocellular carcinoma, markedly reduced adenoma numbers in the AOM-DSS mouse model of CRC and was well tolerated. Levels of pY-STAT3 were increased in the epithelium and stroma of dysplastic colonic mucosa compared to normal colonic mucosa in AOM-DSS mice and adenoma numbers correlated with pY-STAT3 levels in the epithelium and stroma of colonic mucosa. TTI-101 was detected at pharmacologically relevant levels in the plasma and colons of TTI-101-treated AOM-DSS mice and was concentrated within colon tissue. Of note, plasma TTI-101 levels were inversely correlated to pY-STAT3 levels. Importantly, TTI-101 normalized the colon transcriptome of AOM-DSS mice and reduced the expression of STAT3- and STAT1-upregulated genes associated with CRC oncogenesis.

The TTI-101 treatment of mice was started after the development of adenomas to focus on its potential benefit for adenoma and potentially adenocarcinoma treatment. Thaker et al. [49] demonstrated, using the AOM-DSS model, that multiple polyps obstruct the lumen of the distal colon at 50 days in the AOM-DSS model, with the number of tumors peaking between 60 days and 80 days [50]. We started TTI-101 treatment at 57 days after AOM administration, near the expected peak of adenoma formation, and terminated the experiment at 84 days after AOM administration, which was 28 days after the start of TTi-101 treatment. The marked reduction in adenoma numbers in the TTI-101-treatment group indicates a clear treatment effect but may also reflect a preventative effect on adenomas occurring late in this model.

There were 154 candidate genes (upregulated genes) identified whose RNA were increased by the addition of DSS to AOM that were decreased by TTI-101 treatment, including 19 genes shown to be STAT3 gene targets. Importantly, all 19 STAT3-regulated genes are associated with CRC in patients; 16 of the 19 genes have been shown to mediate CRC progression and metastases and to be associated with a worse disease prognosis. Also among the 154 upregulated genes are 9 genes shown to be linked to STAT1 signaling; 6 of the 9 also are linked to STAT3 signaling, while 3 are linked solely to STAT1. The finding that STAT1 upregulates a few CRC-promoting genes is consistent with a growing number of recent observations that STAT1, in addition to its well-known tumor suppressor functions, can promote tumorigenesis, especially in solid tumors [51]. Importantly, the mRNA of all nine of these upregulated genes was decreased by TTI-101. We previously demonstrated that TTI-101 targets STAT1, in addition to STAT3, in human radioresistant head and neck squamous cell carcinoma cell lines [11] and that it also targets STAT5 in a mouse model of increased breast cancer risk caused by pregnancy [52]. Of note, unlike STAT3 and STAT1, a transcriptomic signature related to STAT5 was not found within the 154 upregulated gene set.

TTI-101 is an orally bioavailable inhibitor of STAT3 [11,53,54,55,56,57] first identified in the Tweardy lab using computer-based docking and lead-compound optimization strategies. TTI-101 targets the pY-peptide binding pocket within the Src-homology (SH) 2 domain of STAT3 [11,53,58] and was shown to target STAT3 in mouse models for inflammation, fibrosis, and cancer [11,53,56,59,60,61,62,63,64,65,66]. Extensive pre-clinical evaluations of TTI-101 looking for off-target and on-target adverse effects revealed a high degree of selectivity of TTI-101 for STAT3 [11,59] and evidence of the cross-targeting of STAT1 and STAT5, in certain instances. Previous research, such as twenty-eight day pharmacotoxicology studies in rats and dogs revealed no toxicity, which enabled a Phase I study in solid tumor patients that demonstrated safety, the ability to hit targets in tumors, and a signal of efficacy [67]. Phase II studies of TTI-101 are underway as treatment for hepatocellular carcinoma and for idiopathic pulmonary fibrosis. The studies reported here suggest the possibility that IBD patients with CRC may also benefit from TTI-101 treatment.

## 4. Material and Methods

### 4.1. AOM-DSS Mouse Model of Colitis-CRC (Figure 1)

Eighty (80) six-week-old C57BL/6 male mice were randomized into 4 groups (20 mice per group). Two groups received AOM (10 mg/kg IP once), followed one week later by 3 cycles of DSS treatment, with each cycle consisting of 1 week of 0.5% DSS in drinking water followed by 2 weeks of regular water, as described in [10]. One group received TTI-101 (50 mg/kg formulated in Labrasol 60%; Gatte Fosse, Saint-Priest, France/PEG-400 40%; Spectrum Chemicals, New Brunswick, NJ, USA) by oral gavage, OG; while the other group received vehicle (Labrasol; 60%/PEG-400 40%) daily, starting 57 days after AOM administration when CRC is known to be present. The other two groups consisted of mice treated with AOM alone without DSS followed by treatment with or without TTI-101. Daily clinical evaluations included body weight, water consumption, stool consistency, presence of blood in the stool, and animal behavior. On day 84, mice were humanely euthanized by exsanguination. Blood was anticoagulated, separated into cells and plasma by centrifugation, and used to measure TTI-101 drug levels. The colon was washed with sterile 1×PBS three times, Swiss rolled, formalin-fixed, and paraffin-embedded (FFPE) or snap frozen. FFPE blocks were sectioned for H&E staining and immunohistochemistry (IHC) staining for pY-STAT3. The number of intestinal adenomas/adenocarcinomas were determined by histopathological evaluation of H&E stained 5-micron sections of the complete Swiss roll (5–9 sections per roll). Snap-frozen tissue was used for protein and RNA extraction. Protein extracts were examined for levels of pY-STAT3 and beta-tubulin protein using Luminex bead-based assays. RNA was examined using RNA-seq.

### 4.2. Measurement of TTI-101 in Plasma and Colons

Plasma collected as above and lysates of sections of frozen colons were mixed with stabilizer solution containing 20 mg/mL of NaF, 25 mg/mL of Na_2_SO_3_, and 25 mg/mL of L-ascorbic acid in diH_2_O, with 1×PBS at 1:1 ratio. Aliquots of plasma and lysates were spiked with 5 μL of deuterated TTI-101 (D7) as the internal standard (IS) to a final concentration of 5 μg/mL. Calibration standards and QC samples of TTI-101 were prepared by spiking 5 μL of the relevant working solutions of TTI-101 and SI into 100 μL of blank normal plasma or lysates. TTI-101 was extracted through one-step liquid–liquid extraction (LLE) using methyl tert-butyl ether (MTBE). Samples were reconstituted in 100 μL of methanol before analysis. LC-MS/MS analysis was performed on a QTRAP 5500 Sciex hybrid quadrupole-linear ion trap system with a turbo ion spray source coupled to a Sciex LC Exion liquid chromatography system (Sciex, Framingham, MA, USA). Data acquisition and quantification were conducted using Analyst 1.6. (Redwood City, CA, USA). Chromatographic separation was achieved using a Synergi™ 4 µm Fusion-RP 80 Å, LC Column 50 × 2 mm at a temperature of 40 °C, with a 3 min linear gradient at 500 μL/mL. The aqueous mobile phase (solvent A) was created as follows: 0.1% (*v*/*v*) formic acid and 5 mM ammonium acetate in diH_2_O. The organic phase (solvent B) was 0.1% formic acid and 5 mM ammonium acetate in methanol. Multiple reaction monitoring (MRM) in positive mode (ESI+) was used to detect TTI-101 and an internal standard (IS; TTI-101-d7). The ion pairs for the MRM were *m*/*z* 472.093  >  301.1 for TTI-101 and *m*/*z* 479.200 > 301.1 for IS. The calibration curve for TTI-101 was generated from the peak area ratio of TTI-101 to the peak area of the IS using linear regression analysis with 1/X weight over the range of 0.03–30 μM. All LC-MS/MS reagents including methanol, water, ammonium acetate, and formic acid were obtained from Honeywell Fluka (Morris Plains, NJ, USA). Methyl tert-Butyl Ether (MTBE) was obtained from Sigma Aldrich (St. Louis, MO, USA). The C18 Synergi™ 4 µm Fusion-RP 80 Å LC column (50 × 2 mm) was purchased from Phenomenex (Torrance, CA, USA).

### 4.3. IHC Staining and Digital Image Scoring for pY-STAT3

Sections of Swiss-rolled colon FFPE blocks were deparaffinized by treating with freshly reconstituted xylene thrice (10 min each incubation), followed by washes with 100%, 90%, 80%, and 70% ethanol (10 min each treatment). The slides were then rehydrated with 1×PBS (3 treatments; 5 min each treatment) and then quenched for peroxidase by treatment with 3.0% hydrogen peroxide (prepared in water) for 5 min at room temperature. Slides were immediately washed twice (10 min each wash) with 1×PBS and stained using a primary rabbit monoclonal antibody against pY-STAT3 (D3A7, XP^®^ Rabbit mAb, Cat no. #9145, Cell Signaling Technology, Beverly, MA, USA). Titration studies demonstrated optimum performance at a dilution of 1:200 using the automated bond polymer refine detection kit (cat #DS 9800, Leica Biosystems, Buffalo Grove, IL, USA). This mAb has been used previously by us for both IHC [53] and immunoblotting [12], which confirmed its specificity for the pY705-peptide epitope of STAT3, as reported by the manufacturer and by other groups.

For digital image scoring, pY-STAT3 IHC stained slides were scanned at 20X in Aperio AT2 scanner (Leica Biosystems, Wetzlar, Germany); digital whole slide image (WSI) files in scan scope virtual format were generated using HALO Indica Labs image analysis platform (version 3.5.3577.108). For regions of interest (ROI) selection, histological areas with adenoma/dysplasia and normal-appearing colonic mucosa were identified by a qualified pathologist (LHZ) and then 3–5 ROIs (HALO-squares, 2.2 mm^2^ each) were selected (Figure 3A–G) and segmented into epithelium and stroma (lamina propria) areas of interest (AOI) using either manual annotation tools or aided by a supervised and trained machine learning algorithm (classifier/random forest). Hemorrhage, necrosis, lymphoid follicles, folds, and other artifacts were excluded using the “exclusion annotation” tool. The AOA for quantification of pY-STAT3 were as follows: normal epithelium (N_E), normal stroma (N_S), dysplasia epithelium (D_E), and dysplasia stroma (D_S)**.** The cytonuclear v 2.0.9 algorithm was used for the quantification of nuclear pY-STAT3 levels. Staining and morphological parameters including nuclear contrast, optical density, roundness, segmentation, and level of staining were tuned in real time to achieve optimal performance. Marked-up images were generated and supervised by the pathologist (LHZ) and scored for positive nuclear pY-STAT3 staining intensity: blue (0+), yellow (1+), orange (2+), and red (3+). The pY-STAT3 H-score was determined by totaling the % of cells with each staining level times the staining level: % of cells with staining intensity 0 × 0, plus the % of cells with staining intensity of 1 × 1, plus the % of cells with staining intensity of 2 × 2, plus the % of cells with staining intensity of 3 × 3. The lowest H-score is 0 and the highest achievable H-score is 300.

### 4.4. Total RNA Isolation, RNA Sequencing, and Analysis

Total RNA was isolated from snap-frozen colons by homogenization using reagents provided in the NucleoSpin RNA isolation kit (Cat no. 740955.5, MACHEREY-NAGEL, Düren, Germany). The quality and quantity of the RNA were assessed using Nanodrop one spectrophotometer (Thermoscientific, Waltham, MA, USA). RNA sequencing was performed by Novogene (Sacramento, CA, USA). PCA was performed using the pheatmap R package (Version 4.5.4.1). Differentially expressed genes (DEG) were identified by Novogene using proprietary software (https://www.novogene.com/) (Novomagic, Sacramento, CA, USA) and the following criteria: *p*-value ≤ 0.05 and log fold change ≥1 and ≤1. Heat maps of DEGs were also generated using the pheatmap R package; DEGs were further analyzed using ingenuity pathway analysis (IPA) software Version 1.23.01 (Ingenuity Systems, Redwood City, CA, USA).

### 4.5. Statistical Analyses

All data were verified for normality and similar variances between groups. Student’s *t*-test and Pearson’s correlation were used to compare normally distribution data; Mann–Whitney U-test and Spearman correlation were used to compare data that is not normally distributed. Analyses were performed using R software version 3.6.0 and Prism version 7.0 (GraphPad Software); *p* values <0.05 were considered statistically significant.

## 5. Conclusions

Our group discovered TTI-101, a small-molecule STAT3 inhibitor, that targets the phosphotyrosyl (pY) peptide-binding pocket within the STAT3 SH2 domain, thereby blocking STAT3′s interaction with its pY-peptide ligand on cytokine-activated receptors and inhibiting cytokine-mediated STAT3 activation. We evaluated the efficacy of TTI-101 in the AOM-DSS mouse model of colitis-associated CRC and found that TTI-101 markedly reduced colon tumor burden, at least in part, by normalizing the STAT3-driven pro-oncogenic transcriptome in colonic mucosa. These studies suggest the potential for using TTI-101 to treat CRC in IBD patients with CRC.

## Figures and Tables

**Table 1:** List of genes whose mRNA was increased in vehicle-treated AOM-DSS mouse colons and decreased by TTI-101 treatment.

Gene Symbol	Vehicle vs. Sham (Log 2-Fold Change)	TTI-101 vs. Vehicle (Log 2-Fold Change)	Role in CRC
*Rps8-ps1*	1.204	−1.028	No known role (NKR)
*Camkv*	3.702	−2.824	NKR
*Lypd8l*	2.378	−1.572	NKR
*Pglyrp4*	5.719	−3.344	NKR
*Notum*	6.265	−2.615	NKR
*Ccl2*	1.629	−1.124	Progression/Metastasis
*Tnfrsf11b*	2.706	−1.606	NKR
*Abhd3*	1.536	−1.608	NKR
*Gvin1*	1.518	−1.100	NKR
*Cxcl3*	6.815	−3.389	NKR
*Disc1*	1.258	−1.248	NKR
*Acsbg1*	2.345	−1.431	NKR
*Plat*	2.306	−1.851	NKR
*S100a9*	6.150	−3.654	Progression/Metastasis
*Tgm1*	1.964	−1.774	NKR
*Marcksl1*	1.348	−1.075	NKR
*Acod1*	2.280	−1.609	NKR
*Krt90*	7.013	−2.177	NKR
*Il1b*	2.833	−2.191	Progression/Metastasis
*Trgc2*	1.257	−1.067	NKR
*Alb*	3.817	−3.145	NKR
*Sbspon*	2.174	−1.507	NKR
*Rpl27-ps1*	1.788	−1.222	NKR
*Gpr31a*	3.664	−3.313	NKR
*Cers3*	2.892	−1.623	NKR
*Nkd1*	1.888	−1.182	NKR
*Dmgdh*	2.114	−1.887	NKR
*R3hdml*	3.963	−2.108	NKR
*Prox1os*	2.718	−3.405	NKR
*Arhgdig*	1.196	−1.010	NKR
*Ppbp*	3.807	−2.576	NKR
*Fam89a*	1.682	−1.255	NKR
*Pzp*	5.635	−3.136	NKR
*Timp1*	1.865	−1.530	Progression/Metastasis
*S100a8*	5.627	−3.581	NKR
*Cbx3-ps8*	2.339	−2.428	NKR
*Ahsg*	2.228	−2.551	Progression/Metastasis
*Spock2*	2.297	−1.976	NKR
*Alox12e*	2.745	−2.427	NKR
*Rnf212*	1.096	−1.129	NKR
*Dsc3*	1.850	−1.413	NKR
*Lef1*	1.605	−1.221	Progression/Metastases
*Ascl4*	5.100	−1.815	NKR
*Ppp1r36*	2.161	−2.010	NKR
*Dusp2*	1.076	−1.084	NKR
*Asprv1*	2.488	−1.274	NKR
*Il1rl1*	3.288	−2.043	NKR
*Rpl19-ps3*	3.993	−1.718	NKR
*Halr1*	2.994	−2.124	NKR
*Col17a1*	1.742	−2.141	NKR
*Sox11*	2.312	−3.116	Protection
*Inhbb*	1.006	−1.308	NKR
*Mogat2*	1.495	−1.056	NKR
*Chil1*	1.165	−1.754	NKR
*Lypd3*	3.423	−2.823	NKR
*Slc44a5*	1.611	−1.283	NKR
*Dnmt3c*	2.471	−3.466	NKR
*Pdgfrl*	3.191	−2.332	NKR
*Rab37*	1.412	−1.256	NKR
*Lyz1*	4.044	−2.348	Progression/Metastases
*Prr18*	2.295	−1.354	NKR
*Ccl17*	3.056	−1.452	Progression/Metastases
*Sp5*	3.816	−2.491	NKR
*Ncmap*	1.396	−1.151	NKR
*Dsg3*	2.043	−1.326	NKR
*Crybb1*	2.315	−2.580	NKR
*Klrb1*	1.837	−1.168	NKR
*Alox12*	2.685	−1.417	NKR
*Slc26a9*	4.146	−2.985	NKR
*Il1r2*	2.459	−1.225	NKR
*Rpl3-ps2*	1.597	−1.247	NKR
*Gdf11*	1.316	−1.222	NKR
*Tbx3os2*	2.779	−2.150	NKR
*Prox1*	1.818	−1.335	NKR
*Rpl5-ps2*	1.828	−1.078	NKR
*Klra5*	3.047	−3.180	NKR
*Krt23*	1.139	−1.098	NKR
*Aif1l*	2.406	−2.026	NKR
*Tspan32*	1.684	−1.850	NKR
*Fam162b*	1.123	−1.687	NKR
*St3gal5*	1.001	−1.227	NKR
*Slc9a4*	1.903	−1.143	NKR
*Gata5*	1.780	−1.615	NKR
*Wfdc18*	3.577	−2.419	NKR
*Tmprss11d*	4.894	−5.240	NKR
*Xcl1*	1.802	−2.270	Progression/Metastases
*Trim29*	2.802	−1.303	NKR
*Lect2*	5.616	−4.433	NKR
*Krt5*	5.604	−5.745	NKR
*Aox4*	5.572	−3.349	NKR
*Upk3a*	5.927	−3.336	NKR
*Usp18*	2.294	−1.235	Progression/Metastases
*Tnfsf11*	1.265	−1.774	Progression/Metastases
*Trbc2*	1.491	−1.031	NKR
*Ly6d*	2.856	−2.295	NKR
*Klrb1a*	1.845	−2.020	NKR
*Ntf5*	2.638	−2.056	NKR
*Smim3*	1.458	−1.145	NKR
*Slc30a2*	3.086	−2.196	NKR
*Batf3*	1.043	−1.240	NKR
*Rpl7a-ps11*	1.302	−1.014	NKR
*Bpifb5*	3.232	−3.769	NKR
*Nkg7*	1.515	−1.697	NKR
*Wif1*	3.845	−2.663	NKR
*Cd3g*	1.157	−1.223	NKR
*Prss56*	11.002	−6.618	NKR
*Prom2*	1.500	−1.907	NKR
*Reg3g*	3.918	−4.756	NKR
*Cd3d*	1.484	−1.002	NKR
*Ptpro*	1.279	−1.183	NKR
*Rpl30-ps8*	1.255	−1.745	NKR
*Trbv2*	3.593	−4.271	NKR
*Cxcl9*	2.395	−1.727	Progression/Metastases
*Trgc1*	1.715	−1.434	NKR
*Ltf*	4.096	−3.293	Protection
*Svopl*	1.613	−1.845	NKR
*Ccdc146*	2.126	−2.022	NKR
*Rpl5-ps1*	1.201	−1.291	NKR
*Sema7a*	1.292	−1.274	NKR
*Scgb2b20*	3.004	−3.324	NKR
*Erich2*	1.527	−1.446	NKR
*Psenen-ps*	1.069	−1.482	NKR
*Cd244a*	1.594	−1.089	Immunosuppression
*Cited1*	3.753	−3.005	NKR
*Adam8*	1.110	−1.273	NKR
*Reg3b*	4.126	−6.043	Protection
*Scgb1b3*	1.552	−2.435	NKR
*Esyt3*	2.903	−2.166	NKR
*Col9a3*	3.994	−3.797	NKR
*Lgals2*	1.817	−1.878	NKR
*Ifitm1*	1.988	−1.670	NKR
*Adam28*	2.704	−4.919	NKR
*Adamts3*	1.099	−1.199	NKR
*Mab21l3*	1.613	−2.450	NKR
*Stx11*	1.188	−1.374	NKR
*Ceacam12*	2.372	−2.891	NKR
*Rpl27-ps3*	1.092	−1.204	NKR
*Morc1*	2.447	−3.211	NKR
*Bpifc*	2.966	−5.168	NKR
*Muc6*	2.216	−1.503	NKR
*Scgb2b15*	1.595	−2.288	NKR
*Nanos1*	1.309	−1.468	NKR
*Zap70*	1.656	−1.609	NKR
*Gulo*	4.935	−4.635	NKR
*Mmp7*	5.399	−4.059	Progression/Metastases
*Hmox1*	1.292	−1.175	Progression/Metastases
*Mt3*	2.209	−1.979	NKR
*Sprr2h*	2.384	−4.243	NKR
*Rasl11a*	1.062	−1.257	NKR
*Tesc*	1.592	−1.848	NKR
*Rbm11*	2.405	−1.898	NKR
*Myl7*	3.235	−2.275	NKR
*Fzd10*	1.186	−1.961	NKR
*Akr1c18*	2.300	−3.300	NKR

## Data Availability

The array data will be uploaded onto the NCBI GEO database.
